# What's new in KNIME?

**DOI:** 10.1186/1758-2946-4-S1-F2

**Published:** 2012-05-01

**Authors:** Thorsten Meinl

**Affiliations:** 1 University of Konstanz, Box 712, 78457 Konstanz, Germany

## 

KNIME (Konstanz Information Miner, [1]) is a user-friendly and comprehensive open-source data integration, processing, analysis, and exploration platform. From day one, KNIME has been developed using rigorous software engineering practices and is used by professionals in both industry and academia in over 60 countries.

In the presentation we will show some of the new features in KNIME 2.4 and give an outlook to KNIME 2.5. We also present the KNIME Community Contributions [2], where research groups can easily provide their KNIME extensions to the community.
